# Preparation and Comprehensive Characterization of a Calcium Hydroxyapatite Reference Material

**DOI:** 10.6028/jres.109.042

**Published:** 2004-12-01

**Authors:** Milenko Markovic, Bruce O. Fowler, Ming S. Tung

**Affiliations:** American Dental Association Foundation, Paffenbarger Research Center, National Institute of Standards and Technology, Gaithersburg, MD 20899, USA; National Institute of Dental and Craniofacial Research, NIH, Craniofacial and Skeletal Diseases Branch Research Associate Program at the National Institute of Standards and Technology, Gaithersburg, MD 20899, USA; American Dental Association Foundation, Paffenbarger Research Center, National Institute of Standards and Technology, Gaithersburg, MD 20899, USA

**Keywords:** chemical analysis, crystal size, crystallinity, hydroxyapatite, infrared, morphology, preparation, Raman, solubility, surface area, thermal analysis, unit-cell parameters, x-ray diffraction

## Abstract

Numerous biological and chemical studies involve the use of calcium hydroxyapatite (HA), Ca_10_(PO_4_)_6_(OH)_2_. In this study detailed physicochemical characterization of HA, prepared from an aqueous solution, was carried out employing different methods and techniques: chemical and thermal analyses, x-ray diffraction, infrared and Raman spectroscopies, scanning and transmission microscopies, and Brunauer, Emmett, and Teller (BET) surface-area method. The contents of calcium (Ca^2+^), phosphate (PO_4_^3−^), hydroxide (OH^−^), hydrogenphosphate (HPO_4_^2−^), water (H_2_O), carbonate (CO_3_^2−^), and trace constituents, the Ca/P molar ratio, crystal size and morphology, surface area, unit-cell parameters, crystallinity, and solubility of this HA were determined. This highly pure, homogeneous, and highly crystalline HA is certified as a National Institute of Standards and Technology (NIST) standard reference material, SRM 2910.

## 1. Introduction

Calcium hydroxyapatite (HA), Ca_10_(PO_4_)_6_(OH)_2_, is an important inorganic material in biology and chemistry [1–3]. Biological apatites, which are the inorganic constituents of bone, tooth enamel and dentin, are typically very variable in their composition and morphology, and contain different impurities (Mg^2+^, K^+^, Na^+^, CO_3_^2−^, HPO_4_^2−^, Cl^−^, F^−^, etc.) [1]. In general, these impure biological apatites are designated as calcium deficient or non-stoichiometric apatites.

Synthetic HAs are frequently used as reference materials in biomineralization and biomaterial studies. The composition, physicochemical properties, crystal size and morphology of synthetic apatites are extremely sensitive to preparative conditions. Common impurity phases in synthetic apatites prepared by precipitation from supersaturated aqueous solutions are calcium phosphate compounds such as amorphous calcium phosphates (ACP) with variable compositions of Ca_3_(PO_4_)_2−2_*_x_*(HPO_4_)_3_*_x_* · *n*H_2_O, octacalcium phosphate (OCP), Ca_8_(HPO_4_)_2_(PO_4_)_4_ · 5H_2_O, and calcium hydrogenphosphate dihydrate (DCPD), CaHPO_4_ · 2H_2_O. In addition, the incorporation of various ions as trace impurities (hydrogenphosphate, carbonate, silicate ions, etc.) is very difficult to prevent in any preparative procedure of HA [3].

For control and reference purposes, it is important to have available pure and stoichiometric HA, or nearly stoichiometric HA, characterized in detail with respect to its chemical composition and numerous other important properties. To meet this need, a large amount of highly pure, homogeneous and highly crystalline HA was synthesized by precipitation from aqueous solution of calcium hydroxide and phosphoric acid and then rigorously characterized by chemical and thermal analyses, infrared (IR) and Raman spectroscopies, powder x-ray diffraction (XRD), scanning and transmission microscopies, and surface area and solubility product [4] measurements. The chemical composition and other analyzed properties of this HA qualify it as a standard reference material (NIST, SRM 2910) [5] and it is hereafter denoted as HA-SRM.

Synthetic HA occurs in two structural forms, hexagonal and monoclinic, which have minor structural differences [2]. The hexagonal HA form is usually formed by precipitation from supersaturated solutions at 25 °C to 100 °C and the monoclinic form of HA is primarily formed by heating the hexagonal form at 850 °C in air and then cooling to room temperature [6]. The overall XRD patterns of hexagonal and monoclinic HA are almost identical; however the pattern of monoclinic HA has additional weak lines whose intensities are less than 1 % of the strongest hexagonal HA line [7]. The HA-SRM analyzed here is composed of the hexagonal form (mass fraction of about 75 %) and of the monoclinic form (mass fraction of about 25 %) as determined by normalized additional XRD measurements of the weak line of monoclinic HA at 2*θ* = 36.28° [6–8]. Only the hexagonal form, the major component in HA-SRM, is discussed in this paper. Preparation and characterization of the monoclinic form of HA and differences between the hexagonal and monoclinic HA will be discussed in a separate paper [8].

## 2. Experimental Section

### 2.1 Preparation

Calcium hydroxyapatite-standard reference material (HA-SRM) was synthesized by solution reaction of calcium hydroxide and phosphoric acid in accordance with the preparation of McDowell et al. [9]. In brief, about 5 L of distilled water was boiled for 60 min in a 7.5 L Teflon-coated pot equipped with an electric stirring paddle, a reflux condenser with a CO_2_-absorbing NaOH trap to protect from atmospheric CO_2_, and ports for introducing titrant and nitrogen gas. Calcium oxide (prepared from calcium carbonate heated for 3 h at 1100 °C) was added to the water. Phosphoric acid (concentration 2 mol/L) was added to the calcium oxide/calcium hydroxide slurry at a rate of 0.3 mL/min to 0.6 mL/min and to a final Ca/P molar ratio of 1.67. The reacting mixture was boiled for 2 d. The precipitated solid phase was allowed to settle, the supernatant decanted, and an equal volume of boiled distilled water was added. This suspension was boiled for another 2 d. These washing and boiling procedures were repeated four times until the pH of the supernatant was ≈6; at pH 6, any possible traces of anhydrous dicalcium hydrogenphosphate (DCPA) are converted into HA. The precipitate, collected by filtration, was thoroughly washed with acetone, and then dried at 105 °C for 1 d. The yield was about 1 kg.

### 2.2 Characterization

The HA-SRM was characterized using different methods and techniques. Twenty randomly selected samples were analyzed for both calcium and total phosphorus content. Four samples were analyzed for the content of phosphorus in the form of hydrogenphosphate (HPO_4_^2−^). Fourteen samples were analyzed for water content. Twelve samples were analyzed for carbonate content. The contents of silicon and other trace constituents were determined in one sample. The specific surface area was determined on twelve samples. In addition, scanning and transmission electron microscopy (SEM and TEM), x-ray diffraction (XRD), and infrared (IR) and Raman spectroscopies were employed for detailed characterization.

### 2.3 Chemical Analyses

#### 2.3.1 Calcium Content

Calcium was determined by atomic absorption spectroscopy with a Perkin-Elmer Model 603 spectrophotometer^1^ using an air-acetylene flame and the 442.7 nm wavelength line. Standard calcium solutions used for calibration contained weighed amounts of calcium carbonate (NIST SRM 915, dried at 250 °C for 2 h) and LaCl_3_ in the concentration of about 4.08 mmol/L (about 1000 ppm). The calcium solutions were placed in volumetric flasks (Class A) having volume of 500 mL ± 0.2 mL (later assumed as a standard uncertainty). For experimental details see Refs. [4] and [10].

#### 2.3.2 Phosphorus Content

Phosphorus was determined colorimetrically [11] as the phosphovanadomolybdate complex with a Cary Model 219 spectrometer using a wavelength of 420 nm. Standard phosphate solutions (flasks Class A with volume of 100 mL ± 0.08 mL) used for calibration contained weighed amounts of potassium dihydrogenphosphate (Baker Ultrex Reagent, dried at 105 °C for 2 h) and vanadomolybdate reagent. For experimental details see Refs. [4] and [10].

#### 2.3.3 Hydrogenphosphate Content

The Gee and Deitz method [11] with some modifications [12] was used for determination of the content of phosphorus in the form of hydrogenphosphate (HPO_4_^2−^) in HA. The HA-SRM sample was heated at 550 °C in air for 24 h to convert the hydrogenphosphate into pyrophosphate (P_2_O_7_^4−^). One portion (A) of this heated sample (≈9 mg) was dissolved in 1 mol/L HClO_4_ (in 100 mL volumetric flask) and heated in a boiling water bath for 3 h to hydrolyze the whole content of P_2_O_7_^4−^ into phosphate ions (PO_4_^3−^). Another portion (B) of heated HA-SRM (≈9 mg) was freshly dissolved at 25 °C just prior to phosphate analysis to minimize hydrolysis of P_2_O_7_^4−^ to phosphates. The phosphorus concentrations were determined in both samples as described in Section 2.3.2. The difference in phosphorus contents between samples B and A corresponds to the content of P_2_O_7_^4−^ in the heated HA-SRM and to the content of HPO_4_^2−^ in the unheated HA-SRM sample.

#### 2.3.4 Water Content

The water content was determined from mass loss by three different procedures: (a) The thermogravimetric analysis (TGA) was performed on five samples all in the temperature range from 30 °C to 850 °C (rate 10 °C/min) in a nitrogen atmosphere. (b) Six powdered samples (mass 200 mg to 500 mg) were heated at 850 °C in air at ≈50 % relative humidity for times ranging from 16 h to 20 h. The samples were weighed after cooling for 5 min in a desiccator at ambient conditions. (c) Three of the powdered samples were pressed into pellets and heated at 1000 °C in a steam atmosphere (100 kPa) for 10 h. These samples were weighed after cooling for 5 min in a desiccator.

#### 2.3.5 Carbonate Content

The carbonate (CO_3_^2−^) content was determined by heating ≈5 g of the HA-SRM sample at 1200 °C to liberate CO_2_ that was collected in an absorption cell containing a lithium hydroxide solution. Carbonates in the absorption-cell were determined by automatic coulometric titration. These analyses were done by Galbraith Laboratories, Knoxville, TN.

#### 2.3.6 Silicate and Trace Elements Content

The content of silicon and 63 other elements were analyzed by inductively coupled plasma mass spectroscopy (IPS-MS) by Galbraith Laboratories, Knoxville, TN.

### 2.4 Transmission and Scanning Electron Microscopy

Transmission electron micrographs were obtained from crystals placed directly onto formvar- and carbon-coated nickel grids, or from crystals that were suspended in solution by brief sonication in pure ethanol. In the latter case, ethanol-suspended crystals were allowed to settle onto the support film after which the ethanol was extracted from the edges of the grid with filter paper. Ultrastructural images of the crystals were recorded by transmission electron microscopy at an accelerating voltage of 80 kV using a JEOL JEM 2000FX-II. The samples for scanning electron microscopy were coated with gold and examined with a scanning electron microscope JEOL 5300.

### 2.5 Surface Area

The surface area was determined by the triple-point BET (Brunauer, Emmett, Teller) method [13] with nitrogen as the adsorbate gas and helium as an inert non-adsorbable carrier. The mole fractions of nitrogen in N_2_/He flowing mixtures were 0.1, 0.2 and 0.3. The sample mass was about 200 mg.

### 2.6 Infrared Spectroscopy

IR transmission and second derivative spectra were recorded with a Perkin-Elmer Model 621 spectrometer and with a Nicolet Magna 550 spectrometer, respectively, from the HA-SRM powder suspended in KBr pellets.

#### 2.6.1 IR Transmission Spectra

IR transmission spectra from 4000 cm^−1^ to 300 cm^−1^ were recorded at 48 °C (temperature in instrument light beam) with a Perkin-Elmer Model 621 spectrometer purged with dry CO_2_-free air. KBr sample pellets were run versus a blank KBr pellet in the reference beam to cancel KBr impurity bands, mainly H_2_O bands. KBr pellets were prepared by mixing (not grinding) the pre ground HA-SRM (0.8 mg and 4.0 mg; particle cluster size ≤ 5 μm composed of crystal sizes of 0.1 μm to 0.5 μm) with 400 mg of IR quality KBr (about 20 μm to 40 μm particle sizes). Grinding the sample and KBr together was avoided to reduce additional moisture adsorption from the ground and smaller KBr particles. The HA-SRM and KBr were mixed in a steel capsule on a mechanical shaker and then pressed in a 13 mm diameter evacuated die under a total force of 53,380 N (12 000 pound-force) for 30 s. One die face was machined nonparallel to the second die face, by about 1°. This nonparallel die face produced a wedge-shaped pellet, which reduced spectral interference fringes (especially important for second derivative spectra described below). Spectral slit widths were about 6 cm^−1^ for wavenumbers above 2000 cm^−1^ and 3 cm^−1^ to 5 cm^−1^ for wavenumbers below 2000 cm^−1^. The wavenumber standard uncertainty, calibrated against standard indene bands [14], was 1 cm^−1^ for sharp bands and several cm^−1^ for broad bands.

The ion charges for infrared and Raman bands of different ions are normally omitted in the text.

#### 2.6.2 IR Second Derivative Spectra

Second derivative spectra of absorbance spectra for the *ν*_3_ and *ν*_4_ PO_4_ bands were obtained in the ranges 1120 cm^−1^ to 1000 cm^−1^ and 670 cm^−1^ to 530 cm^−1^ with a Nicolet 550 Magna spectrometer purged with dry CO_2_-free air. The instrumental and data collection conditions were: deuterated triglycine sulfate detector at room temperature, KBr beam splitter, 1 cm^−1^ resolution, 1000 scans, 0.12 cm^−1^ data spacing, Happ-Genzel apodization, no smoothing of *ν*_3_ PO_4_ absorbance spectrum, 25-point smoothing of *ν*_4_ PO_4_ absorbance spectrum, and Nicolet Omnic software to obtain second derivative spectra of the absorbance spectra. High quality absorbance spectra without interference fringes and with low noise are required to obtain meaningful second derivative spectra. To help achieve this, the following were done: (1) to reduce interference fringes, wedge-shaped KBr pellets were prepared as described above (400 mg, 13 mm diameter with thickness increasing from 1.0 mm to about 1.2 mm across the pellet), (2) to eliminate the introduction of possible fringes in the background spectrum, the background for the sample was obtained from the empty pellet holder (no blank KBr pellet) in the spectrometer; and (3) to increase signal to noise, high sample concentrations and resultant high absorbance values of about 1.5 were used; the pellets contained 0.24 mg and 1.0 mg of HA-SRM for *ν*_3_ PO_4_ and *ν*_4_ PO_4_ spectra, respectively. KBr has no bands or impurity bands in the investigated regions. The second derivative wavenumber positions for the *ν*_3_ and *ν*_4_ PO_4_ bands were determined with a standard uncertainty of 0.1 cm^−1^.

### 2.7 Raman Spectroscopy

Raman spectra were recorded with a Spex Model 1401 spectrometer in the 4000 cm^−1^ to 50 cm^−1^ region using the 488.0 nm wavelength excitation from an argon ion laser and a power of 320 mW measured at the sample. Spectra were obtained from about 4 mg of sample powder that was tamped in a cylindrical well (2.5 mm diameter, 1 mm deep) in the center of an aluminum disk 1.5 mm thick and 13 mm in diameter followed by pressing under a sufficient force of about 71,170 N (16,000 pound-force) for 5 s to reduce disk thickness, constrict the sample well and compact the sample. The exciting radiation, upward and vertical, was focused on the compacted sample in the disk tilted about 30° from the incoming radiation direction. Scattered radiation was collected at 90° to the incoming beam direction and detected by a RCA C31034 photomultiplier cooled to −25 °C.

The scattered radiation from the sample was passed through a 488.0 nm filter^2^ placed ahead of the spectrometer entrance slit to reduce the intensity of the 488.0 nm exciting line that was reflected from the opaque sample. This filter markedly reduced the intensity of the 488.0 nm line (about 10^−4^ % of original); this enabled obtaining spectra to within about 50 cm^−1^ of the exciting line and also eliminated spurious “grating ghost” bands.

The spectral slit width was 3.5 cm^−1^. The wavenumber standard uncertainty was ≈0.5 cm^−1^, based on calibration using standard neon emission lines [15] from a neon lamp.

The baseline (BL) was obtained by reflecting the 488.0 nm line from a piece of rough surface platinum foil placed in the normal sample position. One spurious band was observed in the BL at 468 cm^−1^.

### 2.8 X-Ray Diffraction

The x-ray diffraction (XRD) patterns of the powdered HA-SRM samples (about 150 mg in an aluminum holder) were obtained in the range of 3° 2*θ* to 70° 2*θ* with a Rigaku DMAX 2200 diffractometer operating at 40 kV and 40 mA, producing graphite-monochromatized CuKα radiation with wavelength *λ* = 0.15405945 nm, and at a scan speed of 0.030° 2*θ*/min. The relative intensities were determined as diffraction line heights relative to the most intense line normalized to the intensity of 100, with the Materials Data, Inc., JADE 6.1 XRD Patterns Processing software (MDI JADE 6.1).

For determination of diffraction line positions (2*θ*-values), two samples were prepared by mixing HA-SRM with pre ground silicon (Silicon Powder 2*θ*/*d*-Spacing Standard, NIST SRM 640b) that served as an internal standard to correct 2*θ*-values of HA-SRM. The samples contained mass fractions of 88 % HA-SRM and 12 % silicon. Two separate scans with the speed of 0.012° 2*θ*/min were obtained for each sample. For each scan, the position of each HA-SRM and silicon diffraction line was determined with MDI JADE 6.1 as the average of four measurements using pseudo-Voigt and Pearson-VII profile functions (two measurements for each profile function).

The HA-SRM unitcell (lattice) parameters were calculated with the Least Squares Unit Cell Refinement and Indexing for Personal Computer (LSUCRIPC) program^3^; the input data were 2*θ*-values and corresponding indices (hkl) of the eight diffraction lines in the range from 39° 2*θ* to 54° 2*θ*, which have relative intensities above 10, and do not overlap with other HA-SRM and silicon diffraction lines. For each HA-SRM sample, the unit-cell parameters were calculated from the average 2*θ*-values determined from the two separate scans. The final HA-SRM unit-cell parameters are the average of the data for the two samples.

Diffraction theory predicts that the diffraction lines of a XRD powder pattern will be very sharp for a crystalline material consisting of sufficiently large and strain-free crystallites [16]; therefore, the XRD line broadening (peak width) inversely correlates with crystal size and lattice perfection. The term “crystallinity” is commonly used to represent the crystallite size and lattice perfection. For determination of diffraction line angular width at its half-height, the lines having *hkl* indices (200), (002), (102), (210), (310) and (004) were recorded earlier with a vertically mounted Rigaku Denki diffractometer system operating at 40 kV and 25 mA, producing graphitemonochromatized CuKα radiation with wavelength *λ* = 0.15405945 nm (time constant 10, scale 500 counts/s, scan speed 0.03125° 2*θ*/min). The diffraction line angular width, *B*, at its half-height above background was measured with an optical magnifier and expressed in ° 2*θ*. The angular width (*B*) was corrected for instrumental line broadening (*b*) caused by instrument imperfections [16]. The corrected value of the angular width (*β*) expressed in ° 2*θ*, was calculated from Warren’s equation [16]

$$\beta ={\left(\beta^{2}-b^{2}\right)}^{1/2}.$$

A stoichiometric, highly crystalline monoclinic hydroxyapatite (hc-HA) prepared by solid-state thermal reaction [17] was used as a reference substance in determination of the value of *b* (the angular width at the half-height of hc-HA diffraction lines). The *b*-values for hc-HA diffraction lines were determined for the same six lines as for HA-SRM.

The reciprocal of the *β* value (1/*β*) correlates to the crystallite size/perfection [16].

### 2.9 Statistical Analysis

Uncertainties were assessed by the CIPM (International Committee for Weights and Measures) approach [18]. The uncertainty of a measurement result commonly consists of several components. An estimated standard deviation called a standard uncertainty, *u*_i_, represents a component of uncertainty. A combined standard uncertainty, *u*_c_, was computed by the method of propagation of uncertainties [18,19] and represents at the level of one standard deviation the combined effects of all standard uncertainties, *u*_i_’s. According to the CIPM recommendation [18] the uncertainty of a measurement result is expressed with expanded uncertainty, *U*. Results in this paper, except as noted, are expressed as mean value ± *U*, where *U* = 2*u*_c_.

## 3. Results and Discussion

### 3.1 Chemical Composition

#### 3.1.1 Calcium

The mass fraction of calcium in HA-SRM varied from 38.78 % to 39.49 % with a mean value of 39.15 % ± 0.10 % (Table 1).

#### 3.1.2 Phosphorus

The mass fraction of the total phosphorus content in HA-SRM varied from 18.111 % to 18.235 % with a mean value of 18.181 % ± 0.037 %.

#### 3.1.3 Ca/P Molar Ratio

From the mean values of Ca and P contents the calculated Ca/P molar ratio was 1.664 ± 0.005. This value is in agreement with the ratio of 1.6649 ± 0.0005 independently determined for this HA-SRM by thermal-product analysis [6,8]. The Ca/P ratio of 1.664 for this HA-SRM is about 0.2 % below the stoichiometric value of 1.6667.

#### 3.1.4 Hydrogenphosphate and Phosphate

The mass fraction of phosphorus present in the form of hydrogenphosphate ions (HPO_4_^2−^) was 0.191 % ± 0.010 % and accordingly, the mass fraction of HPO_4_^2−^ was 0.592 % ± 0.030 % (Table 1). The mass fraction of phosphorus present in the form of phosphate ions (PO_4_^3−^) is the difference between mass fractions of the total phosphorus content (18.181 % ± 0.037 %) and of phosphorus present as HPO_4_^2−^ (0.191 % ± 0.010 %), giving the mass fraction of phosphorus present as PO_4_^3−^ of 17.99 % ± 0.05 %. From this value the calculated mass fraction of PO_4_^3−^ was 55.16 % ± 0.15 % (Table 1). The contents of PO_4_^3−^ and HPO_4_^2−^ expressed as molar fractions of the total phosphate content were 98.95 % and 1.05 %, respectively.

#### 3.1.5 Water

The total mass loss (expressed as the mass fraction) of samples heated continuously from 30 °C to 900 °C in a nitrogen atmosphere was 1.70 % ± 0.05 %. This mass loss is primarily attributed to water loss based on water band intensity changes in the IR spectrum of HA-SRM heated at 105 °C and 850 °C. The HA-SRM water content is the difference between the mass fractions of the total mass loss (1.70 % ± 0.05 %) and the water loss derived from hydrogenphosphate pyrolysis into pyrophosphate and thermal reaction of calcium pyrophosphate and HA forming *β*-tricalcium phosphate; the calculated mass fraction of water derived from these thermal/chemical reactions was 0.111 % ± 0.006 %. Therefore, the mass fraction of water in HA-SRM was 1.59 % ± 0.05 % or 0.902 H_2_O molecule per HA-SRM unit cell (Table 1).

**Fig. 1:**
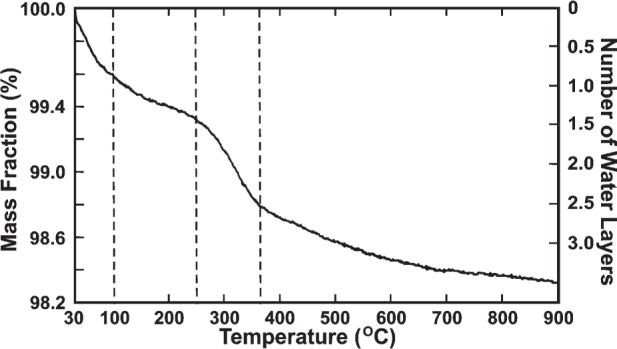
TG-curve for HA-SRM in the temperature range from 30 °C to 900 °C in a nitrogen atmosphere. The left ordinate denotes the mass fraction and the right ordinate gives the corresponding calculated number of water layers progressively removed from the HA-SRM surface.

The TG-curve for HA-SRM (Fig. 1) is shown in the temperature range from 30 °C to 900 °C; on the left ordinate is mass fraction and on the right ordinate is the corresponding calculated number of water layers progressively removed from the HA-SRM surface. The number of water layers on the HA-SRM surface was calculated from the HA-SRM surface area of 18.3 m^2^/g (Section 3.2) and a cross-sectional area of 0.115 nm^2^ for an adsorbed water molecule [20] on the HA surface; one monolayer of water corresponds to the mass fraction of 0.47 %. Rootare and Craig [20] have carried out detailed studies of vapor phase adsorption of water on HA. They found that the water monolayer that is in contact with the HA surface (chemisorbed layer) was more strongly bound than the additional water layers (all physisorbed layers) that involved water/water contacts only. To completely remove the chemisorbed monolayer, heating at 300 °C in vacuum was required whereas the physisorbed layers could be removed at 20 °C in vacuum.

The TG-curve (Fig. 1) showed an initial mass loss (expressed as mass fraction) of ≈0.4 % in the temperature range from 30 °C to 100 °C and a mass loss of ≈0.3 % in the range from 100 °C to 250 °C. These two losses (mass fractions), giving a sum of ≈0.7 %, correspond to ≈1.5 layers mainly of physisorbed water although some chemisorbed water is also expected to be lost between 100 °C and 250 °C [20]. Between 250 °C and 360 °C, a loss of ≈0.55 % was observed which corresponds to ≈1 layer of chemisorbed water. This temperature range, 250 °C to 360 °C, and mass loss equivalent to ≈1 water layer are consistent with data of Rootare and Craig [20] for the chemisorbed water layer. The mass fraction lost in the temperature range from 360 °C to 850 °C was ≈0.45 %. Of this ≈0.45 %, ≈0.11 % corresponds to water loss from HPO_4_^2−^/P_2_O_7_^4−^/HA/*β*-TCP reactions, ≈0.02 % corresponds to loss from CO_3_^2−^ decomposition on heating to 850 °C and the remainder of ≈0.32 % corresponds to ≈0.7 layer of water that is more strongly held by the crystals than the chemisorbed layer.

From these TG-data it appeared that the total number of water layers at the surface of the HA-SRM crystals was ≈2.5; ≈1.5 layers correspond to physisorbed water and ≈1 layer to chemisorbed water. The location of the more strongly-held water, equivalent to ≈0.7 layer or about one water molecule per 5.6 HA-SRM unit cells is uncertain. It may be “structural” water or water trapped within crystals.

The mass fraction of water in HA-SRM determined from mass loss of powdered HA-SRM samples heated in air at 850 °C for 16 h to 20 h, then cooled in a desiccator and weighed in the laboratory atmosphere (50 % relative humidity) at ambient temperature was 1.430 % ± 0.034 %, whereas the mass fraction of water in HA-SRM determined in samples pressed into pellets and heated in a steam atmosphere at 1000 °C for 10 h and then cooled and weighed as above was 1.564 % ± 0.028 %. In both cases the HA-SRM water content was lower than in the samples heated and weighed in the nitrogen atmosphere because of fast readsorption of surface water during cooling and weighing in the air atmosphere at ambient temperature.

#### 3.1.6 Carbonate

Carbonate ions are a common impurity in HA. The mass fraction of carbonate found in HA-SRM was in the range from 0.029 % to 0.033 % with the mean value of 0.032 % ± 0.002 % (Table 1). This carbonate content corresponds to 0.00545 CO_3_^2−^ ion per HA-SRM unit cell (Table 1) or to one CO_3_^2−^ ion per 183 HA-SRM unit cells.

#### 3.1.7 Silicate

The mass fraction of silicon of 0.015 % (Table 2) expressed as mass fraction of silicate ions, SiO_3_^2−^, was 0.0406 % (Table 1). This content corresponds to 0.00546 SiO_3_^2−^ ion per HA-SRM unit cell or to one SiO_3_^2−^ ion per 183 HA-SRM unit cells. The source of the silicon impurity was most plausibly the boro-silicate glass apparatus used in preparation of HA-SRM.

#### 3.1.8 Trace Constituents

Trace constituents with mass fractions above 0.0005 % (>5 ppm) in HA-SRM are listed in Table 2 and summarized in Table 1. Approximately 0.001 atom each of Al, B, Mg, Na and Sr occurs per HA-SRM unit cell (Table 2), which corresponds to approximately one of each atom per 1000 unit cells. The sum of trace constituent atoms of 0.00595 per HA-SRM unit cell (Table 1) corresponds to one trace constituent atom per 168 HA-SRM unit cells.

#### 3.1.9 Hydroxide

In Table 1 are listed the contents of analyzed HA-SRM constituents: calcium, phosphate, hydrogenphosphate, water, carbonate, silicate and sum of trace constituents. From these contents the number of constituents per HA-SRM unit cell was calculated by normalizing the total number of phosphate groups (PO_4_^3−^ and HPO_4_^2−^) to six, 5.937 PO_4_^3−^ and 0.063 HPO_4_^2−^. The relative charge attributed to the total number of hydroxide ions (OH^−^) per unit cell was calculated from the difference between positive and negative relative charges of all unit-cell constituents; a mean value of −2.026 for OH^−^ ions balanced the total charge to zero. This calculated number of 2.026 ± 0.070 of OH^−^ ions per HA-SRM unit cell corresponds to the mass fraction of 3.37 % ± 0.12 % of OH^−^ in HA-SRM (Table 1).

#### 3.1.10 Sum of Mass Fractions

The total sum of mass fractions of all constituents was 99.95 % ± 0.22 % (Table 1); this shows high accuracy of the chemical analyses.

### 3.2 Crystal Morphology and Specific Surface Area

**Fig. 2:**
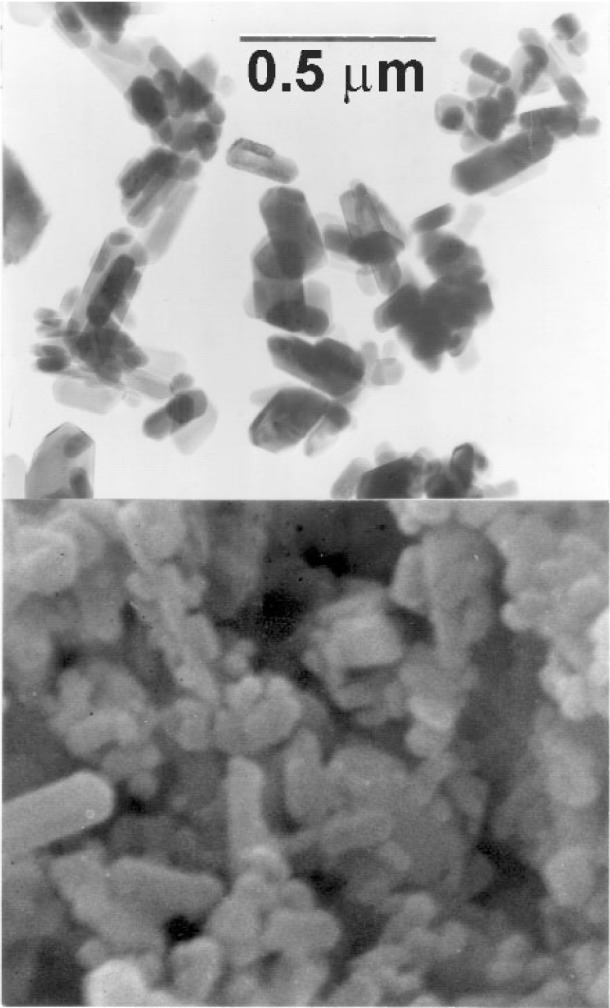
Transmission electron micrograph (top) and scanning electron micrograph (bottom) of the HA-SRM crystals. Both micro-graphs have the same magnification and the bar length in the top micrograph is 0.5 μm.

Transmission and scanning electron micrographs of the HA-SRM crystals are shown in Fig. 2. Generally, the crystals appear to have a cylindrical shape with heights of ≈0.1 μm to 0.3 μm and diameters of ≈0.05 μm to 0.15 μm. The specific surface area determined by BET was 17.7 m^2^/g to 19.1 m^2^/g with an average value of 18.3 m^2^/g ± 0.3 m^2^/g. This specific surface area for the HA-SRM crystals compares well with the value of 16.6 m^2^/g calculated by assuming an average cylindrical particle with height of 0.2 μm and diameter of 0.1 μm.

The HA sample of McDowell et al. prepared by precipitation from solutions had a specific surface area of 16.7 m^2^/g determined by BET [9]. This value of 16.7 m^2^/g is in agreement with the above value of 18.3 m^2^/g for HA-SRM. These data indicate the reproducibility of crystal sizes and surface area of HAs prepared by the same method.

### 3.3 Infrared Spectra

#### 3.3.1 IR Transmittance Spectra

**Fig. 3:**
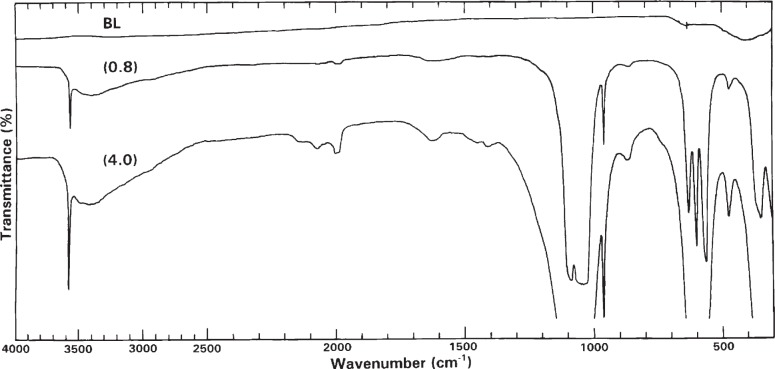
Infrared percent transmittance spectra of HA-SRM from concentrations of 0.8 mg and 4.0 mg of HA-SRM/400 mg of KBr in the 4000 cm^−1^ to 300 cm^−1^ region. BL denotes the KBr pellet baseline.

IR transmittance spectra of HA-SRM at two different concentrations (0.8 mg and 4.0 mg HA-SRM per 400 mg KBr) are shown in the 4000 cm^−1^ to 300 cm^−1^ range in Fig. 3. The spectra show the bands of HA along with additional bands that are ascribed to impurity ions (CO_3_^2−^, HPO_4_^2−^, and silicate ions), and associated H_2_O.

Bands of HA [21]: (a) The bands at 3572 cm^−1^, 631 cm^−1^, and 342 cm^−1^ arise from stretching, librational, and translational modes, respectively, of OH^−^ ions. (b) The 1090 cm^−1^ and about 1040 cm^−1^ bands arise from *ν*_3_PO_4_, the 962 cm^−1^ band arises from *ν*_1_ PO_4_, the 601 cm^−1^ and 574 cm^−1^ bands arise from *ν*_4_ PO_4_, and the 472 cm^−1^ band arises from *ν*_2_ PO_4_. (c) The group of weak intensity bands in the 2200 cm^−1^ to 1950 cm^−1^ region derives from overtones and combinations of the *ν*_3_ and *ν*_1_ PO_4_ modes. The sharpness of bands, especially sharpness of the 631 cm^−1^, 601 cm^−1^, and 574 cm^−1^ bands, indicate a well-crystallized HA.

Bands of CO_3_^2−^ impurity ions: The weak intensity bands at about 1410 cm^−1^ and 1450 cm^−1^ in the spectrum of HA-SRM at high concentration (4.0 mg of HA-SRM per 400 mg KBr) are attributed to components of the *ν*_3_ mode of a trace amount of CO_3_^2−^. The mass fraction of CO_3_^2−^ in HA-SRM determined by chemical analysis, Sec. 3.1.6, was 0.032 %. The areas and intensities of these CO_3_ bands correspond to mass fraction of about 0.03 % CO_3_^2−^ by comparison to CO_3_ bands of other HA samples [6] containing chemically analyzed CO_3_^2−^ mass fractions of about 0.3 %; this band intensity agreement for this low CO_3_^2−^ content helps identify these weak intensity bands as CO_3_ bands. Bands of other CO_3_ modes, *ν*_4_ and *ν*_1_, were not detected because of their weak intensities and the *ν*_2_ CO_3_ band at about 872 cm^−1^, with intensity about one fifth that of *ν*_3_ CO_3_, is obscured by the HPO_4_ band at 875 cm^−1^. The CO_3_ bands at 1410 cm^−1^ and 1450 cm^−1^ derive from CO_3_^2−^ (designated the “B-type” carbonate) that replace PO_4_^3−^ ions in the HA lattice [22] (and references therein). Bands at 1455 cm^−1^ and about 1540 cm^−1^, which derive from CO_3_^2−^ (designated the “A-type” carbonate) that replace OH^−^ ions in the HA lattice [23], were not detected. The mass fraction of 0.032 % chemically determined CO_3_^2−^, corresponds to one CO_3_^2−^ ion per 1101 total phosphate ions (PO_4_^3−^ and HPO_4_^2−^).

Bands of HPO_4_^2−^ impurity ions: The band at 875 cm^−1^ is attributed to arise from HPO_4_^2−^ ions for several reasons [24,25]. Chemical analysis shows that HA-SRM contains 1.05 HPO_4_^2−^ ions per 98.95 PO_4_^3−^ ions (Sec. 3.1.4, Table 1) or molar fraction of 1.05 % HPO_4_^2−^ with respect to the total P content. The isolated HPO_4_^2−^ ion has 9 predicted infrared active internal modes for its highest symmetry point group, C_3v_, and 12 predicted infrared active modes for its lowest symmetry point group, C_1_. At this very low molar fraction of 1.05 % HPO_4_^2−^, of the 9 to 12 possible bands, only the 875 cm^−1^ band is clearly detectable; the other HPO_4_ bands are obscured by the PO_4_ bands of HA and, in addition, the (-O-H) bands of the HOPO_3_^2−^ ions are broad and weak in intensity. The normalized intensity and area of the 875 cm^−1^ band correlates with HPO_4_^2−^ content determined by chemical analysis. A HA sample containing a HPO_4_^2−^ molar fraction of 2.34 % by chemical analysis [3,6] had a 875 cm^−1^ normalized band area 2.1 times that of the HA-SRM that contained HPO_4_^2−^ molar fraction of 1.05 % determined by chemical analysis. In addition, this 875 cm^−1^ HPO_4_ band was, as expected, missing in spectra of HA-SRM that had been heated at 550 °C because of condensation of HPO_4_^2−^ ions to form P_2_O_7_^4−^ ions and H_2_O.

Bands of silicate impurity ions: The mass fraction of Si in HA-SRM determined by chemical analyses was 0.015 % (Sec. 3.1.7); the mass fraction calculated as the SiO_3_^2−^ was 0.0406 % (Table 1). Previous work [6] on other HAs prepared by precipitation in glass apparatus from solution at 100 °C and high pH produced HAs that contained Si mass fraction of about 0.1 % to 0.3 % determined by chemical analyses. IR spectra of these HAs had weak bands, not deriving from HA, at 890 cm^−1^, ≈830 cm^−1^, ≈750 cm^−1^ and ≈500 cm^−1^ and a Raman band at 890 cm^−1^ whose intensities correlated with silicon content. Consequently, these bands were attributed to silicate ions, and their most probable source was the glass apparatus. The type of silicate ion SiO_3_^2−^ (chain or ring structures), Si_2_O_7_^6−^, or SiO_4_^4−^ in these HAs was not identified with certainty by IR or Raman methods primarily because of the low silicate contents and resultant weak band intensities along with interference from the strong HA bands. Nevertheless, the combined IR and Raman data and additional thermal data suggested that (SiO_3_^2−^)_3_ = Si_3_O_9_^6−^ ring and Si_2_O_7_^6−^ ions may be present and SiO_4_^4−^ and acidic silicates less probable. The high concentration spectrum of HA-SRM in Fig. 3 has very weak bands at 890 cm^−1^ and ≈750 cm^−1^; these two bands are better discerned in the high concentration spectrum of the heated HA-SRM that will be shown in the paper on monoclinic HA [8]. These 890 cm^−1^ and 750 cm^−1^ bands are attributed to silicate ions and are assumed to be SiO_3_^2−^ ions.

Bands of H_2_O molecules: The broad band from about 3700 cm^−1^ to 2500 cm^−1^ derives from the *ν*_3_ and *ν*_1_ stretching modes of hydrogen-bonded H_2_O molecules, and the band at 1630 cm^−1^ derives from the *ν*_2_ bending mode of the H_2_O molecules. The thermogravimetric data in Table 1 show a mean mass loss (expressed as mass fraction) of 1.59 % on heating HA-SRM that is primarily attributed to loss of adsorbed water. In the IR spectra of HA-SRM after heating at 850 °C [8], the above water bands are, as expected, missing; this indirectly identifies H_2_O as the principal component lost on heating.

#### 3.3.2 IR Second Derivative Spectra

**Fig. 4:**
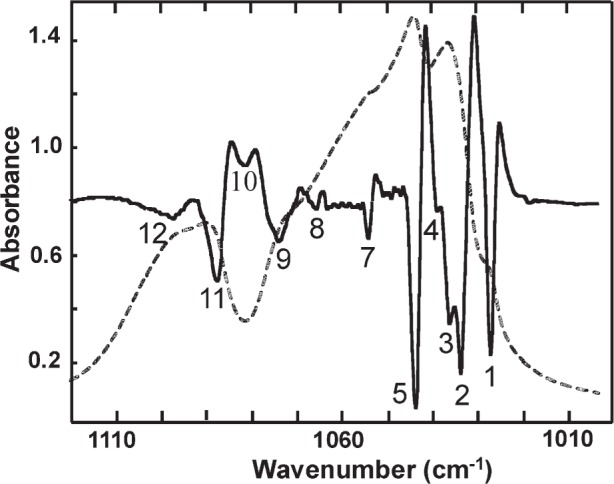
Infrared absorbance spectrum of the *ν*_3_ PO_4_ mode of HA-SRM (dashed line) and second derivative of the absorbance spectrum (solid line). The second derivative spectrum ordinate scale, not shown, is arbitrary. The second derivative spectrum was adjusted to full ordinate range and the minima denoted by numbers identify band positions in the absorbance spectrum.

**Fig. 5:**
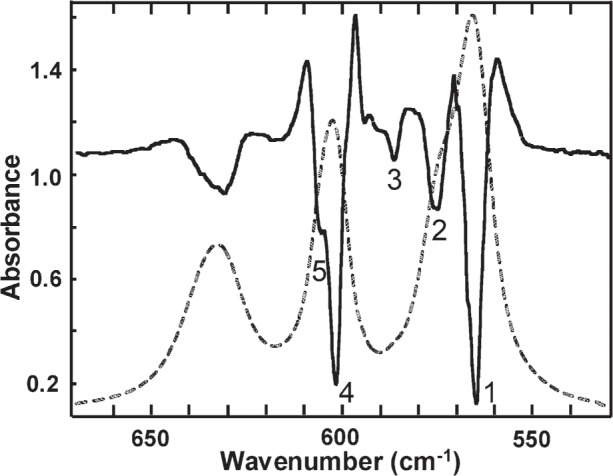
Infrared absorbance spectrum of the *ν*_4_ PO_4_ mode of HA-SRM (dashed line) and second derivative of the absorbance spectrum (solid line). The absorbance band at 633 cm^−1^ and second derivative band at 633 cm^−1^ derive from the OH^−^ librational mode. The description of the second derivative spectrum is the same as that given in Fig. 4.

IR second derivative spectra of the *ν*_3_ and *ν*_4_ PO_4_ bands are shown in Fig. 4 and Fig. 5, respectively, and the second derivative band positions are given in Table 3. Second derivative spectra of the *ν*_1_ and *ν*_2_ PO_4_ bands are not shown. Only one *ν*_1_ PO_4_ band was detected at 962.9 cm^−1^ in second derivative spectra and the instrument detector response, progressively lower in the 500 cm^−1^ to 400 cm^−1^ region along with the weak *ν*_2_ PO_4_ band intensity, precluded obtaining well-resolved second derivative spectra of the *ν*_2_ PO_4_ band although the bands occur at about 474 cm^−1^ and 462 cm^−1^. Under 1 cm^−1^ resolution, eleven *ν*_3_ PO_4_ bands were resolved (Fig. 4). Two of these bands, numbered 3 and 4 in Fig. 4 and in Table 3, are attributed to arise from the mass fraction of about 25 % of monoclinic HA; these bands will be discussed in the paper on monoclinic HA [8]. Thus, nine bands were detected for the *ν*_3_ PO_4_ mode of this hexagonal HA-SRM. In Fig. 5, five second derivative *ν*_4_ PO_4_ bands were detected; the absorbance band and second derivative band at 633 cm^−1^ derive from the OH^−^ librational mode.

### 3.4 Raman Spectra

**Fig. 6:**
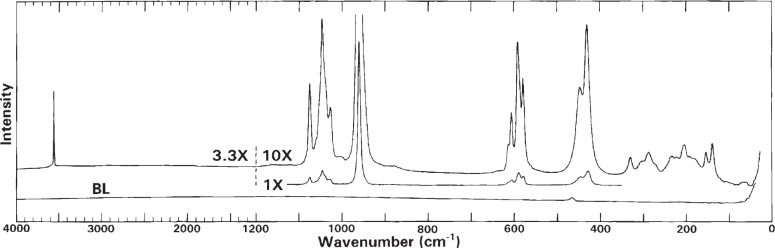
Raman spectra of HA-SRM from 4000 cm^−1^ to 50 cm^−1^ recorded at relative intensities of 1 and 10 below 1200 cm^−1^ and 3.3 above 1200 cm^−1^. BL denotes the baseline.

Raman spectra of HA-SRM in the range from 4000 cm^−1^ to 50 cm^−1^ recorded with relative intensities of 1 and 10 in the range below 1200 cm^−1^ and with relative intensity of 3.3 in the range above 1200 cm^−1^ are shown in Fig. 6. The spectra have the bands of hexagonal HA and two additional bands that arise from HPO_4_^2−^ impurity ions. Under the spectral resolution used (spectral slit width of 3.5 cm^−1^), no bands of monoclinic HA are resolved.

Bands of HA [26–29]: (a) The 3573 cm^−1^ and 329 cm^−1^ bands arise from stretching and translational modes of the OH^−^ ions, respectively; the OH^−^ librational bands expected in the 630 cm^−1^ region are not clearly detected although two bands are predicted by C_6_ factor group symmetry analysis [21]. (b) The 1076 cm^−1^, 1052 cm^−1^ (shoulder, sh), 1047 cm^−1^, 1040 cm^−1^ (sh), and 1028.5 cm^−1^ bands arise from *ν*_3_ PO_4_, the very strong 962 cm^−1^ band arises from *ν*_1_ PO_4_, the 614 cm^−1^, 607 cm^−1^, 590 cm^−1^, and 579 cm^−1^ bands arise from *ν*_4_ PO_4_, and the 447 cm^−1^ and 431 cm^−1^ bands arise from *ν*_2_ PO_4_. (c) The group of weak intensity bands in the 329 cm^−1^ to 50 cm^−1^ region derives from translations of the Ca^2+^, PO_4_^3−^, and OH^−^ ions and librations of the PO_4_^3−^ ions. The 329 cm^−1^, 305 cm^−1^, and 270 cm^−1^ bands have been assigned to vibrations of the 2[(Ca_II_)_3_-(OH)] sublattice of hexagonal HA, and the band at 285 cm^−1^ primarily to libratory phosphate motions [28,30].

Bands of CO_3_^2−^ impurity ions: The strongest intensity CO_3_ band, *ν*_1_, for the B-type CO_3_^2−^ impurity occurs at 1070 cm^−1^; this band is obscured by the strong intensity PO4 band at 1076 cm^−1^. The other CO3 modes *ν*_3_, *ν*_4_, and *ν*_2_ (*ν*_2_ is expected to be Raman active because of low symmetry of CO_3_^2−^ ion) have band positions not obscured by the PO_4_ bands, but they have weak intensities and were not detected. The *ν*_1_ CO_3_ band for A-type CO_3_^2−^, unobscured by PO_4_ bands, occurs at 1106 cm^−1^ [31], and is useful for detecting the A-type CO_3_^2−^. However, this band was absent; this was expected because the IR spectra did not have bands for the A-type CO_3_^2−^.

Bands of HPO_4_^2−^ impurity ions: The weak band at 1005 cm^−1^ is assigned to symmetric stretching of the HPO_4_^2−^ ions and the weak band at 880 cm^−1^ to [P-(OH)] stretching of the HPO_4_^2−^ ions [6,32]. Similarly as in IR spectra, these two Raman bands increase in intensity with an increase in HPO_4_^2−^ content, and they are missing in spectra of HA-SRM that had been heated at 550 °C because of thermal conversion of HPO_4_^2−^ ions to P_2_O_7_^4−^ ions. This independent detection of HPO_4_^2−^ ions in Raman spectra confirms the IR data on HPO_4_^2−^ ions.

Bands of silicate impurity ions: Bands of the trace silicate impurity, probably present as SiO_3_^2−^ or Si_2_O_7_^6−^ ions, were not detected because of the low silicate mass fractions (about 0.04 % as SiO_3_^2−^ or Si_2_O_7_^6−^ ions). The mass fraction of 0.2 % of silicate impurity (as Si_2_O_7_^6−^) was detectable in other HA preparations by the 890 cm^−1^ band arising from Si_2_O_7_^6−^ ions.

Bands of H_2_O molecules: Water vibrational modes give rise to weak intensity stretching and bending bands in Raman spectra. The water component in HA-SRM (mass fraction of 1.59 %) causes IR bands at 3700 cm^−1^ to 2500 cm^−1^ and 1630 cm^−1^; these water bands, expected at about the same wavenumbers in Raman spectra, were not observed in Raman spectra under the spectral intensity expansion used in Fig. 6.

### 3.5 Combined Infrared and Raman Data

A rigorous comparison of the number and coincidences of the IR and Raman bands cannot be made with the present data because equivalent high-resolution second derivative Raman spectra were not obtained for HA-SRM. Although additional Raman bands may be detected, comparisons of the predicted and observed current data are meaningful and are given in Table 4. IR and Raman bands that have wavenumber positions within 2 cm^−1^ were considered coincident.

The number and coincidence or noncoincidence of the infrared and Raman active bands predicted according to factor group analysis for the *ν*_1_, *ν*_2_, *ν*_3_, and *ν*_4_ PO_4_ modes of HA having hexagonal structures (P6_3_/m, C_6h_) and (P6_3_, C_6_) [21] are given in Table 4 along with the observed number and coincidence or noncoincidence of the infrared and Raman bands of HA-SRM. Hexagonal HA belongs to the space group P6_3_; if, however, the OH^−^ ions are disregarded, the overall structure is P6_3_/m. The lower P6_3_ symmetry results from the position, heteronuclearity, and order of the OH^−^ ions. In fluoroapatite (P6_3_/m space group), the F ions are located along the *c*-axis on the mirror planes passing through the Ca_II_ triangles, whereas in hexagonal HA, the OH^−^ ions, with internuclear axes coincident with the *c*-axis, are displaced about 0.03 nm from the planes of the Ca_II_ triangles with protons pointing away from the Ca_II_ triangles [33]; thus, the mirror planes passing through the Ca_II_ triangles are lost and the P6_3_ space group results. These minor structural differences cause considerable differences in the vibrational selection rules.

A comparison of the predicted and observed spectral data for the PO_4_ modes in Table 4 shows a better fit with C_6h_ than with C_6_ symmetry. Weights of 1, 2, 3, and 3 were applied to data for the *ν*_1_, *ν*_2_, *ν*_3_, and *ν*_4_ PO_4_ modes, respectively; these numerical weights correspond to the degeneracy of each mode. About 74 % of the total spectral data for the PO_4_ modes (the total number of IR and Raman bands and the number of coincident/noncoincident bands) better fit with C_6h_ symmetry. About 26 % of the total spectral data for the PO_4_ modes (the total number of IR bands for the *ν*_3_ and *ν*_4_ PO_4_ modes, the coincidence of the *ν*_1_ IR and Raman PO_4_ bands, and the coincidence/noncoincidence of the *ν*_3_ Raman bands) better fit with C_6_ symmetry. This better agreement with C_6h_ symmetry is in accordance with previous conclusions based on fewer spectroscopic data [21,26] that also favored C_6h_ symmetry (P6_3_/m space group) for hexagonal HA.

The number of observed IR *ν*_3_ and *ν*_4_ PO_4_ bands is larger than predicted for C_6h_ symmetry. This is believed to derive from sources other than effects of lower P6_3_ symmetry, and this will be considered in a separate paper [34].

### 3.6 X-Ray Diffraction Pattern

**Fig. 7:**
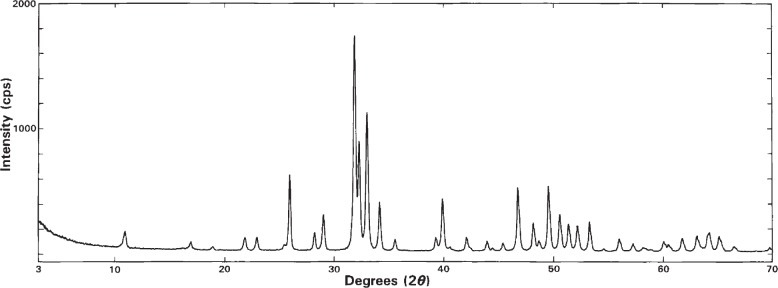
X-ray diffraction pattern of HA-SRM in the 2*θ* range from 3° to 70°.

The XRD pattern of HA-SRM is shown in Fig. 7. The observed positions of diffraction lines (2*θ* and corresponding *d*_2_*_θ_*) and their relative intensities (*I*_rel_) are listed in Table 5. These *d*_2_*_θ_* and *I*_rel_ for HA-SRM are in full agreement with the corresponding values reported for hexagonal HA (JCPDS, Card No. 9-432) [35]. The additional weak lines of monoclinic HA that have relative intensities less than 1 % of the strongest hexagonal HA line were not observed at the intensity scale shown in Fig. 7. The additional XRD measurements, from which a mass fraction of about 25 % of monoclinic HA was determined in HA-SRM, will be reported in a separate paper [8].

### 3.7 Unit-Cell Parameters

The *a* and *c* unit-cell parameters for HA-SRM calculated from the eight selected diffraction lines (2*θ*-values marked with a in Table 5) are listed in Table 6. The complete set of *d*-values (*d*_calc_) calculated from these unitcell parameters is listed in Table 5. These *d*_calc_-values are in excellent agreement with *d*_2_*_θ_*-values determined from the 2*θ*-values that were not used for unit-cell parameters calculation (2*θ*-values without asterisks in Table 5).

The *a* and *c* unit-cell parameters for HA-SRM determined in this paper are in very good agreement with the parameters determined for the same material by the Rietveld analyses [5,36], given in Table 6. The average values of these two independently determined unit-cell parameters for HA-SRM by the Rietveld analyses are: *a* = 0.94235 nm, and *c* = 0.68852 nm. As compared with these average unit-cell parameters, the values determined in this paper are 0.003 % larger in *a*, and 0.003 % larger in *c* than the corresponding average values. The values for similarly prepared hexagonal HA [9] determined by the Rietveld analyses [37] (Table 6, HA-McDowell) are 0.065 % smaller in *a*, and 0.001 % larger in *c* than the corresponding average values for HA-SRM determined by the Rietveld analyses.

### 3.8 Crystallinity

The mean angular widths at half-height (denoted as *B* and *b*) for the (200), (002), (102), (210), (310), and (004) diffraction lines of HA-SRM (*B*-values) and of hc-HA (*b*-values) and the calculated 1/*β* values are listed in Table 7. The 1/*β* values were determined in the next crystal directions: (i) along the *a*-axis perpendicular to *b*-*c* plane, 1/*β* (200) = 6.0 (° 2*θ*) ^−1^ ± 0.3 (° 2*θ*) ^−1^, (ii) along the *c*-axis perpendicular to *a*-*b* plane, 1/*β* (002) = 8.4 (° 2*θ*) ^−1^ ± 0.2 (° 2*θ*) ^−1^ and 1/*β* (004) = 7.1 (° 2*θ*) ^−1^ ± 0.3 (° 2*θ*) ^−1^, (iii) perpendicular to *c*-axis, 1/*β* (210) = 5.8 (° 2*θ*) ^−1^ ± 0.2 (° 2*θ*) ^−1^ and 1/*β* (310) = 5.5 (° 2*θ*) ^−1^ ± 0.2 (° 2*θ*) ^−1^ and (iv) perpendicular to *b*-axis, 1/*β* (102) = 8.2 (° 2*θ*) ^−1^ ± 0.4 (° 2*θ*) ^−1^. The bigger 1/*β* value denotes the larger crystal size and lattice perfection in corresponding crystal directions showing for HA-SRM the biggest 1/*β* values for size/strain in directions along *c*-axis and that perpendicular to *b*-axis, and the smallest 1/*β* values for size/strain in directions along *a*-axis and those perpendicular to *c*-axis. The 1/*β* (002) for HA-SRM is ≈10 % smaller and 1/*β* (310) for HA-SRM is ≈40 % larger than corresponding values for HA prepared by DCPA hydrolysis at pH ≈6.5 [38]. For HA-SRM the ratio of 1/*β* (002) and 1/*β* (310) values, *R*(1/*β*) = [1/*β* (002)]/[1/*β* (310)] = *β* (310)/*β* (002), is 1.6 and for HA hydrolyzed from DCPA at pH ≈6.5 the ratio *R*(1/*β*) is 2.5 [38]. These *R*(1/*β*)-values can be correlated with the ratio of crystal height (longer dimension) and crystal width (shorter dimension) of these HA crystals determined microscopically. HA-SRM crystals for which *R*(1/*β*) = 1.6 have cylindrical shape with the height/diameter ratio of ≈2 (Fig. 2), and HA crystals hydrolyzed from DCPA, for which *R*(1/*β*) = 2.5, have plate-like shape with very large height/width ratio of ≈10 [38]. It indicates that the *c*-axis of these HA crystals is in the direction along the crystal height and the *a*-axis is in the direction along the crystal width.

### 3.9 Solubility

The solubility product of this HA-SRM was previously determined [4]. The saturated solutions with respect to HA-SRM were obtained by dissolution of HA-SRM crystals in aqueous solutions of phosphoric acid for 60 d at 37.0 °C ± 0.1 °C. The thermodynamic solubility product, *K*_sp_, of HA-SRM defined as *K*_sp_(HA) = *a*^5^(Ca^2+^) *a*^3^(PO_4_^3−^) *a*(OH^−^), where *a* denotes ion activity, was calculated from measured equilibrium calcium and phosphate concentrations and pH values as input data. The mean value and standard uncertainty, *u*_i_, of the twelve replicate determinations (*n* = 12) was *K*_sp_(HA) = (2.03 ± 0.04) × 10^−59^. The standard uncertainties, *u_i_*(*y*), derived from other sources were also determined. These other sources were uncertainties in measurements of Ca, P and pH, and uncertainties in dissociation constants of phosphoric acid (*K*_1_, *K*_2_, and *K*_3_) and stability constant of calcium phosphate complexes used for *K*_sp_ calculation. The major contributions to the combined uncertainty, *u*_c_ = 0.356 × 10^−59^, were from pH measurements (*u_i_* = 0.196) and the *K*_3_ literature value (*u_i_* = 0.280). The expanded uncertainty, *U* = 2*u*_c_, was 0.71 × 10^−59^; thus, the thermodynamic *K*_sp_(HA) at 37 °C, expressed as the mean ±*U*, was (2.03 ± 0.71) × 10^−59^ and its p*K*_sp_(HA) was 58.69 ± 0.15. This *K*_sp_(HA) value of (2.03 ± 0.71) × 10^−59^ is in very good agreement with the literature value of (2.36 ± 0.28) × 10^−59^ determined under similar conditions for similarly prepared HA [9].

## 4. Conclusions

The chemical and physical analyses of this HA-SRM are considered very reliable based on the consistency of the combined results. This HA-SRM has application as a standard of numerous well established chemical and physical properties to compare with and to establish the validity of equivalent analyses on natural and synthetic hydroxyapatites, the mineral phases in calcified tissues, and in testing and regulation.

## Figures and Tables

**Table 1:** Chemical composition of calcium hydroxyapatite standard reference material (HA-SRM) along with the calculated number and total relative charge of constituent ions per HA-SRM unit cell^a^

Constituent	Mass fraction (%)	*n*^b^	Number of constituent ions/HA-SRM unit cell^c^	Total relative charge of constituent ions/HA-SRM unit cell^d^
Ca^2+^	39.15 ± 0.10	20	9.985 ± 0.026	+19.970 ± 0.051
PO_4_^3−^	55.16 ± 0.15	20	5.937 ± 0.016	−17.811 ± 0.048
HPO_4_^2−^	0.592 ± 0.030	4	0.063 ± 0.003	−0.126 ± 0.006
H_2_O	1.59 ± 0.05	5	0.902 ± 0.028	0
CO_3_^2−^	0.032 ± 0.002	12	0.00545 ± 0.00034	−0.0109 ± 0.0007
SiO_3_^2−^	0.0406^e^	1	0.00546	−0.0109
Trace elements^f^	0.0181^f^	1	0.00595^f^	+0.0144
OH^−^	3.37^h^ ± 0.12		2.026^h^ ± 0.070	−2.026^g^ ± 0.070

Sum	99.95 ± 0.22 0			0

a All results expressed as mean value ±*U*, where *U* is expanded uncertainty.

b Number of replicate measurements.

c Number of constituent ions normalized to six phosphate groups (5.937 PO_4_ + 0.063 HPO_4_).

d Calculated from relative electrical charge of the constituent ion time number of the constituent ions.

e Calculated from silicon content in Table 2.

F From Table 2.

g Calculated to balance total charge to 0.

h Derived from calculated relative charge of −2.026^g^.

**Table 2:** Contents of trace constituents^a^ and silicon in HA-SRM

Trace constituent	Mass fraction (%)	Number of ions/HA-SRM unit cell^b^
Al^3+^	0.0029	0.00110
Ba^2+^	0.0024	0.00018
B^3+^	0.0015	0.00142
Mg^2+^	0.0029	0.00122
Na^+^	0.0031	0.00138
Sr^2+^	0.0044	0.00051
Zn^2+^	0.0009	0.00014

Sum	0.0181	0.00595

Si	0.0150	0.00546

a Trace constituents having mass fraction >0.0005 % are included.

b Calculated number of ions per unit-cell.

**Table 3:** IR wavenumber positions of *ν*_3_ and *ν*_4_ PO_4_ bands of HA-SRM obtained from second derivative spectra

	PO_4_ bands (cm^−1^)	
Band number^a^	*ν*_3_	*ν*_4_
1	1027.0	565.1
2	1033.7	575.3
3	1036.0^b^	586.4
4	1038.7^b^	601.8
5	1043.6	605.4
6		
7	1054.0	
8	1065.4	
9	1073.9	
10	1081.3	
11	1087.7	
12	1097.5	

a Refer to Figs. 4 and 5.

b These two bands are attributed to arise from monoclinic HA (mass fraction ≈25 %).

**Table 4:** Predicted number and coincidence or noncoincidence of infrared and Raman *ν*_1_, *ν*_2_, *ν*_3_, and *ν*_4_ bands for PO_4_ modes of hexagonal structures (P6_3_/m, C_6h_) and (P6_3_, C_6_) of calcium hydroxyapatite^a^ and observed bands for HA-SRM

Hexagonal structure	Spectra	PO_4_ modes			
		*ν*_1_	*ν*_2_	*ν*_3_	*ν*_4_
P6_3_/m, C_6h_	IR predicted	1nc	2nc	3nc	3nc
	R predicted	2nc	3nc	5nc	5nc
P6_3_, C_6_	IR predicted	2c	4c	6c	6c
	R predicted	1nc, 2c	2nc, 4c	3nc, 6c	3nc, 6c
HA-SRM	IR observed	1c	2nc	7nc, 2c	4nc, 1c
	R observed	1c	2nc	3nc, 2c	3nc, 1c

a Predicted from Ref. 21.

IR = infrared. R = Raman. c = coincident. nc = noncoincident.

**Table 5:** 2*θ*-values and relative intensities (*I*_rel_) observed from the XRD pattern of HA-SRM, *d*-values determined from 2*θ*-values (*d*_2_*_θ_*), *d*-values calculated from unit cell parameters (*d*_calc_), and corresponding indices (*hkl*)

2*θ*(°)	*d*_2_*_θ_* (nm)	*d*_calc_(nm)	*I*_rel_	*hkl*
10.85	0.815	0.816	8	100
16.87	0.525	0.526	3	101
18.84	0.471	0.471	2	110
21.75	0.408	0.408	6	200
22.84	0.389	0.389	6	111
25.35	0.351	0.351	2	201
25.86	0.344	0.344	36	002
28.11	0.317	0.317	8	102
28.92	0.308	0.308	16	210
31.77	0.281	0.281	100	211
32.18	0.278	0.278	47	112
32.90	0.272	0.272	65	300
34.04	0.263	0.263	22	202
35.44	0.253	0.253	5	301
39.18	0.2297	0.2297	6	212
39.793*	0.2263	0.2263	22	310
40.43	0.2229	0.2229	1	221
41.98	0.2150	0.2150	6	311
42.30	0.2135	0.2134	1	302
43.84	0.2063	0.2063	4	113
44.36	0.2040	0.2040	1	400
45.29	0.2000	0.2000	4	203
46.683*	0.1944	0.1944	28	222
48.068*	0.1891	0.1891	12	312
48.58	0.1872	0.1872	3	320
49.458*	0.1841	0.1841	30	213
50.474*	0.1807	0.1807	15	321
51.254*	0.1781	0.1781	11	410
52.061*	0.1755	0.1755	11	402
53.167*	0.1721	0.1721	14	004
54.43	0.1684	0.1684	1	104
55.85	0.1645	0.1645	6	322
57.11	0.1611	0.1611	4	313
58.03	0.1588	0.1588	2	501
58.28	0.1582	0.1582	2	412
58.74	0.1570	0.1570	1	330
59.93	0.1542	0.1542	4	420

* 2*θ*-values have expanded uncertainty (*U*) of ±0.004° 2*θ* (n = 4).

**Table 6:** Unit-cell parameters for HA-SRM and similarly prepared HA by McDowell et al. [9]

Sample	*a*(nm)	*c*(nm)	XRD analysis	Reference
HA-SRM	0.94238 ± 0.00009^a^	0.68854 ± 0.00006^a^	Standard	This paper
HA-SRM	0.942253 ± 0.000013^a^	0.688501 ± 0.000009^a^	Rietveld	[5]
HA-SRM	0.94244 ± 0.00002^b^	0.68854 ± 0.00002^b^	Rietveld	[36]
HA-McDowell	0.94174 ± 0.00002^b^	0.68853 ± 0.00002^b^	Rietveld	[37]

a Mean value ± expanded uncertainty (*U*).

b Mean value ± standard deviation.

**Table 7:** The line width at half-height (*B*-value) for selected XRD lines of HA-SRM, the corresponding line width at half-height (*b*-value) of hc-HA^a^, and calculated 1/*β* values

*hkl*	*B*(° 2*θ*)	b(° 2*θ*)	1/*β* (° 2*θ*) ^−1^
200	0.225 ± 0.007	0.150 ± 0.002	6.0 ± 0.3
002	0.188 ± 0.002	0.145 ± 0.002	8.4 ± 0.2
102	0.183 ± 0.005	0.136 ± 0.004	8.2 ± 0.4
210	0.218 ± 0.003	0.134 ± 0.003	5.8 ± 0.2
310	0.218 ± 0.004	0.120 ± 0.004	5.5 ± 0.2
004	0.181 ± 0.005	0.114 ± 0.003	7.1 ± 0.3

a Highly crystalline HA prepared by solid state thermal reaction [17].

## Footnotes

1 Certain commercial equipment, instruments or materials are identified in this paper to foster understanding. Such identification does not imply recommendation or endorsement by the National Institute of Standards and Technology, the National Institutes of Health, or the ADA Foundation nor does it imply that the materials or equipment identified are necessarily the best available for the purpose.

2 Holographic Notch Plus Filter, Kaiser Optical Systems, Inc., Ann Arbor, MI 48103.

3 Least Squares Unit Cell Refinement, NDSU version Fargo 90.10.13.em after Appleman and Evans (1973), implementation by Roy G. Garvey, Department of Chemistry, North Dakota State University, Fargo, ND 58105-5516.
